# Low-temperature Mössbauer spectroscopy of organs from ^57^Fe-enriched HFE^(−/−)^ hemochromatosis mice: an iron-dependent threshold for generating hemosiderin

**DOI:** 10.1007/s00775-022-01975-y

**Published:** 2022-12-13

**Authors:** Shaik Waseem Vali, Paul A. Lindahl

**Affiliations:** 1 Department of Biochemistry and Biophysics, Texas A&M University, College Station, TX USA; 2 Department of Chemistry, Texas A&M University, College Station, TX 77843-3255 USA

**Keywords:** Thalassemia, Hemosiderin, Magnetic susceptibility, Serum, Hepcidin, Ferroportin, Transferrin, non-transferrin-bound iron, or NTBI, Iron–sulfur clusters, Heme

## Abstract

Hereditary hemochromatosis is an iron-overload disease most often arising from a mutation in the Homeostatic Fe regulator (HFE) gene. HFE organs become overloaded with iron which causes damage. Iron-overload is commonly detected by NMR imaging, but the spectroscopic technique is insensitive to diamagnetic iron. Here, we used Mössbauer spectroscopy to examine the iron content of liver, spleen, kidney, heart, and brain of ^57^Fe-enriched HFE^(−/−)^ mice of ages 3–52 wk. Overall, the iron contents of all investigated HFE organs were similar to the same healthy organ *but from an older mouse*. Livers and spleens were majorly overloaded, followed by kidneys. Excess iron was generally present as ferritin. Iron–sulfur clusters and low-spin Fe^II^ hemes (combined into the central quadrupole doublet) and nonheme high-spin Fe^II^ species were also observed. Spectra of young and middle-aged HFE kidneys were dominated by the central quadrupole doublet and were largely devoid of ferritin. Collecting and comparing spectra at 5 and 60 K allowed the presence of hemosiderin, a decomposition product of ferritin, to be quantified, and it also allowed the diamagnetic central doublet to be distinguished from ferritin. Hemosiderin was observed in spleens and livers from HFE mice, and in spleens from controls, but only when iron concentrations exceeded 2–3 mM. Even in those cases, hemosiderin represented only 10–20% of the iron in the sample. NMR imaging can identify iron-overload under non-invasive room-temperature conditions, but Mössbauer spectroscopy of ^57^Fe-enriched mice can detect all forms of iron and perhaps allow the process of iron-overloading to be probed in greater detail.

**Graphical Abstract:**

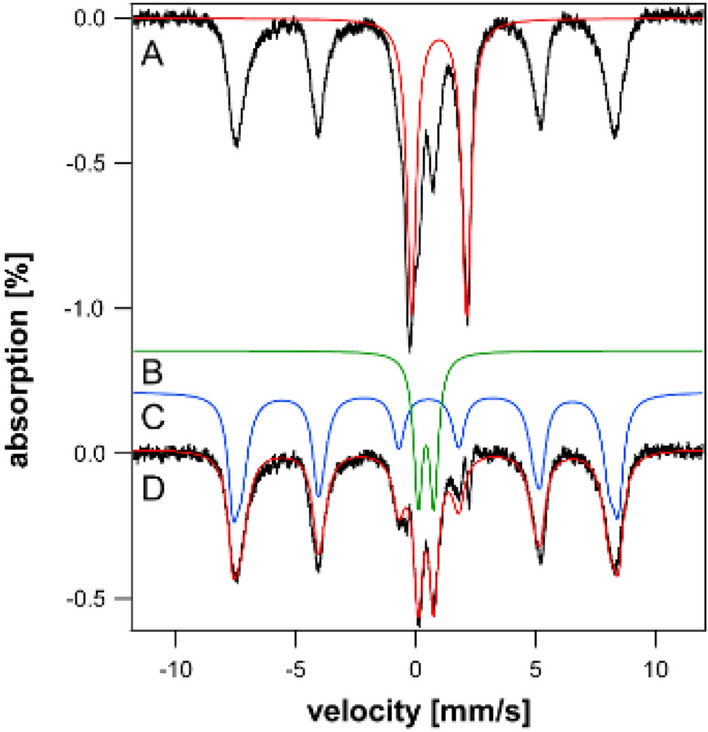

**Supplementary Information:**

The online version contains supplementary material available at 10.1007/s00775-022-01975-y.

## Introduction

Hereditary hemochromatosis is an iron-overload disease affecting 1 in 200 humans of northwestern European ancestry [1, 2]. Its most common form arises from a C282Y mutation of the Homeostatic Fe regulator (HFE) gene. HFE is involved in the biosynthesis of hepcidin, a peptide generated by hepatocytes. Hepcidin controls iron export from enterocytes, which line the basolateral surface of the duodenum, and from reticuloendothelial macrophages, which are found in the spleen, liver, and elsewhere. Hepcidin regulates iron import into the blood by binding and inactivating ferroportin (FPN), a membrane-bound iron export protein. Binding promotes the translocation of FPN to lysosomes where it is hydrolyzed; this halts iron export into plasma.

In healthy individuals, iron that enters the blood binds transferrin (TFN). Transferrin-bound iron is distributed to cells of the body by binding a receptor on the plasma membrane. TFN enters cells via receptor-mediated endocytosis. In healthy individuals, ~ 30% of TFN is holo, while most of the remainder is *apo*. Because both forms are present at significant concentrations, TFN serves as an iron buffer that can absorb a bolus of nutrient iron and also release iron to cells. Hepatocytes in individuals with hemochromatosis produce insufficient hepcidin. This causes excessive nutrient iron to enter the blood and TFN to saturate. Once saturated, additional iron enters plasma as Non-Transferrin-Bound Iron or NTBI which enters organs and overloads them with iron [3].

In individuals with hemochromatosis, the liver is the first organ to become overloaded [4]. Once in cells, the excess iron is converted into either ferritin or hemosiderin [5]. Ferritin is an approximately spherically shaped iron-storage protein with a hollow core that can be filled with Fe^III^ nanoparticles. Hemosiderin is an insoluble, amorphous, and heterogeneous degradation product of ferritin located mainly in secondary lysosomes [6]. Excess iron in the liver can cause fibrosis, cirrhosis, and cancer. Other organs also accumulate iron but generally have a lower iron-binding capacity than the liver. However, they may be more easily damaged by iron accumulation [7, 8].

Cells of the reticuloendothelial system, including Kupffer macrophages in the liver, red-pulp macrophages in the spleen, and central nurse macrophages in the bone marrow, are especially sensitive to iron-overload. These cells recycle most bodily iron for erythropoiesis [9]. Red-pulp macrophages extract iron from senescent erythrocytes by degrading hemoglobin. Excessive iron increases hepatic hepcidin expression which causes macrophages to sequester iron [10]. Iron can deposit in splenic macrophages in the form of hemosiderin [11, 12].

Iron in the HFE kidney accumulates mainly in the medulla. Iron is removed from plasma at the glomerulus associated with Bowman’s capsule and is then reabsorbed in the collecting tubules associated with the Loop of Henle [13–15].

Excessive iron in the heart accumulates in cardiomyocytes where it can cause arrhythmias and congestive heart failure [16, 17]. The heart imports less ferric citrate (injected into rodents as an NTBI mimic) than the liver, likely due to the lower expression levels of the divalent metal importer Zip14 [18, 19]. However, it also imports NTBI through calcium channels [6, 18–22].

The brain accumulates a modest amount of iron in iron-overloaded mice [23–25]. NTBI is imported into the brain of hypotransferrinemic mice [26–28]. Individuals with β-thalassemia, another iron-overload disease, have iron deposits in the anterior pituitary [29]. These deposits can alter the production of hormones and lead to hypogonadism. Radioactive ^59^Fe injected into WT mice was detected in the ventricles within 2 h [30]. Within 24 h, such iron spread throughout the brain, with especially high concentrations localized in the choroid plexus.

Iron-overload in organs is most popularly detected in vivo by an NMR method that is sensitive to superparamagnetic materials such as ferritin [31]. This method, which involves monitoring proton transverse relaxation times (T2*), is widely used, because it is non-invasive [32]. Hocq et al. [33] used NMR spectroscopy to estimate the iron content of the liver, spleen, and brain in four human donors (without iron-overload). Ferritin iron darkens T2-weighted NMR images. Relaxation times on brain, liver, and spleen samples can be measured at different magnetic fields. However, the correlation with total iron content is imperfect. T2* values are affected by the size and density of ferritin iron cores and, more importantly, are insensitive to diamagnetic forms of iron such as [Fe_4_S_4_]^2+^ and [Fe_2_S_2_]^2+^ clusters or as low-spin Fe^II^ hemes.

Low-temperature AC magnetic susceptibility has also been used to investigate iron-overloaded organs. Gutierrez et al. [34] characterized liver, spleen, and heart tissues of DBA/2 HFE knockout mice using this method together with transmission electron microscopy and Selected Area Electron Diffraction to investigate the chemical iron speciation in mice with overload diseases. In that study, iron accumulated in the liver in 9 wk HFE vs WT mice, but no differences between HFE and WT mice spleens and hearts in terms of iron-overload were observed. There was some evidence of ferritin degrading and hemosiderin forming. The same group examined 12 wk HFE and WT mice; HFE mice accumulated ferritin in livers, but the amount of ferritin in kidney and heart was minor and similar for HFE and WT mice.

We have previously used Mössbauer (MB) spectroscopy to examine the iron content of brain [35], liver [36], and heart [37] from ^57^Fe-enriched healthy control mice of various ages. We also examined hearts and livers from 12 wk ^57^Fe-enriched HFE mice (the natural lifespan of mice is ~ 100 wk). For healthy organs, the two dominant iron species observed by MB include ferritin and a combination of [Fe_4_S_4_]^2+^ clusters and low-spin Fe^II^ hemes. The latter two species collectively yield the “central quadrupole doublet”, called the CD (δ = 0.45 mm/s; ΔE_Q_ = 1.15 mm/s) in MB spectra. Viewed simplistically, ferritin reflects iron that is *stored*, whereas the CD reflects iron that is *used*—mainly but not exclusively in mitochondrial respiration. The absolute and relative amounts of ferritin vs. the CD change with the organ and with the age of the mouse. A small contribution due to nonheme high-spin (NHHS) Fe^II^ has also been observed as a quadrupole doublet in MB spectra. NHHS Fe^II^ likely reflects the labile Fe^II^ pool in cells, though other Fe^II^ species in the sample must also contribute.

Healthy newborn livers contain a high concentration of iron, mainly in the form of ferritin [36]. Within the first few weeks of life, much of this iron exits the liver and is delivered to other organs as needed for normal development. At 3–4 wk of age, most iron in the liver is present as the CD. As animals age, ferritin accumulates. After the initial exodus, the iron concentration within liver cells (with blood contributions removed) is only ~ 300 µM. Diseased livers (HFE and IRP2-deficient) contain significantly higher concentrations of iron, in the form of ferritin.

In contrast, the iron content of the heart is dominated by the CD [37]. In fact, young hearts contain little if any ferritin. Ferritin levels in the heart increase with age. Brains from fetuses contain ~ 270 µM Fe, mostly in the form of ferritin [35]. The concentration of iron declines in newborn and young brains (to ~ 120 µM Fe), due to the expanding volume of the developing brain. The iron concentration in adult brains is only ~ 200 µM. With age, more ferritin iron accumulates.

MB spectroscopy has also been used by other groups to characterize the iron content of iron-overloaded organs. Hemosiderin was found to be present and even dominate the iron in iron-overloaded organs. This form of iron can be distinguished from ferritin by comparing spectra collected at 5 K and 60/70 K. At the higher temperatures, magnetic interactions due to ferritin iron collapse, whereas those due to hemosiderin do not [38, 39]. Ferritin exhibits a sextet at ~ 5 K, but by 60–70 K, it exhibits a quadrupole doublet [38, 40]. Seldon et al. [41] concluded that hemosiderin is the dominant form of iron in iron-overload diseases and that it is responsible for organ damage. St. Pierre et al. [42] concurred that hemosiderin (rather than ferritin) damages iron-overloaded organs. The same group collected MB spectra of spleens, pancreas, heart, and livers of β-thalassemia patients [40]. They observed a sextet at 12 K and a broad singlet at 78 K, with parameters of high-spin Fe^III^. They detected hemosiderin in spleens and pancreas, and concluded that there are at least three forms of hemosiderin. Spectra of whole tissues were composed of a superposition of features from hemosiderin and ferritin. Ward et al. [43] found that hemosiderin was the major iron-storage protein in tissues of iron-overloaded individuals; it accounted for three-quarters of the iron in iron-loaded human livers. Webb et al. [44] used MB to examine the pancreas of humans with β-thalassemia, and isolated ferritin and hemosiderin from tissues. Using MB spectroscopy, St. Pierre et al. [45, 46] found that hemosiderin was the major form of iron in spleens from human β-thalassemia patients and as well as iron-overloaded spleens and livers from rodents. Other studies reported a closer balance between the two. Chua-anusorn [39] used 12 K and 60 K MB to examine iron deposits in human thalassemic heart tissue. About 40% of the iron was hemosiderin and 35% was ferritin (20% was unassigned). Gutierrez et al. [47] found that ferritin rather than hemosiderin dominated the iron content of HFE mice.

In our previous studies, we did not observe hemosiderin in any healthy or diseased organ. Rather, we exclusively observed ferritin, ISCs, hemes, and small contributions of NHHS Fe^II^. However, we only examined one liver and one heart from HFE mice, and then only at a relatively young age (12 wk) [37]. Here, we used variable temperature MB spectroscopy to examine livers, hearts, spleens, kidneys, and brains from ^57^Fe-enriched HFE mice of different ages. Organs were perfused with buffer to remove excess blood, dissected under anaerobic conditions, loaded immediately into MB cups, frozen, and evaluated using MB spectroscopy. We characterized the type of iron that accumulated as well as the age-dependence of that accumulation. We provide evidence that hemosiderin is generated when iron in organs exceeds a threshold concentration.

## Experimental procedures

All procedures involving mice were approved by the Animal Use Committee at TAMU (Animal Use Protocol 2018–0204). HFE^(−/−)^ mice, henceforth called HFE mice (stock number 017784, B6.129S6-Hfe < tm2Nca > /J), were purchased, along with control mice (C57BL/6 J) from The Jackson Laboratory (www.jax.org). Animals were housed in the LAAR facility in the School of Veterinary Medicine at TAMU. Mice were raised in disposable all-plastic cages (Innovive model MVX1) containing synthetic bedding (Alpha-Dri Irradiated; Lab Supply, Houston) and all-plastic water bottles. Room temperature was 28 ± 1 °C and lighting was on a 12/12 h cycle. Mice were bred on an iron-deficient mouse diet (TD.80396.PWD; www.envigo.com) spiked with 50 mg ^57^Fe (Cambridge Isotope Laboratories; 95.5% enriched oxide powder) per kg chow. The diet was prepared by spraying 50 mL of each of 4 stock solutions onto the chow powder while mixing in a glass bowl. Each stock solution contained the required concentration of ^57^Fe^III^ citrate (2 × excess of citrate relative to iron) plus sodium ascorbate (5 × relative to iron). The resulting moistened material was pelleted using a plastic pipe and a snug-fitting glass rod. Pellets were pushed out of the pipe onto a glass pan, then baked at 80 °C for 2–4 h. Once cooled, they were sealed in plastic bags and refrigerated until use. Mice were offered food and distilled water ad libitum.

Animals ranging in age from 3 to 52 wk were transported to the Chemistry department at TAMU where they were sacrificed. Mice were anaesthetized by injecting ketamine (5 mg/20 gm mouse) and xylazine (1 mg/20 gm mouse) subcutaneously. Exsanguination was by cardiac puncture once tests for pain (foot-pad squeeze) showed no response. Between 0.3 and 1.2 mL of blood was removed from each animal. Carcasses were imported into a refrigerated N_2_-atmosphere glove box (Mbraun Labmaster 130) containing 1–20 ppm O_2_ where they were perfused by passing Ringers’ buffer into the heart (0.1 ml/min for 5–10 min). Brain, liver, heart, spleen, and kidneys were then removed by dissection. After organ masses were determined, they were placed in Mössbauer cups. The liver from 1 mouse was used per cup, whereas 2 hearts, 1 brain, 2–3 spleens, and 3–4 kidneys were used in single MB cups. Samples were frozen in the box by importing an aluminum block that had been pre-chilled in LN_2_, placing the samples on the block surface, and waiting 1–2 min until they had frozen. Frozen samples were removed from the box and stored in LN_2_. MB Spectroscopy was performed as described [35–37] using WMOSS software for simulations.

Iron concentrations were determined as described [48], with minor modifications. Organs were thawed after collecting MB spectra. Samples were transferred into pre-weighed falcon tubes, and masses were determined. One mL of 70% Trace Metal Grade HNO_3_ (Fisher Chemical) was added per 0.1 g of organ mass. Tubes were sealed using electrical tape and incubated at 80 °C overnight. After cooling, solutions were diluted 250 × using high-purity water in replicates of 3 to a final volume of 5 mL and 2% (v/v) HNO_3_ concentration. A series of calibration standards were prepared with the TEXAM15 stock solution (Inorganic Venture, Christiansburg Virginia, USA), also affording 2% HNO_3_. Fe concentrations of samples and standards were measured by ICP-MS (Agilent 7700x) in He collision mode.

## Results

**Fig. 1:**
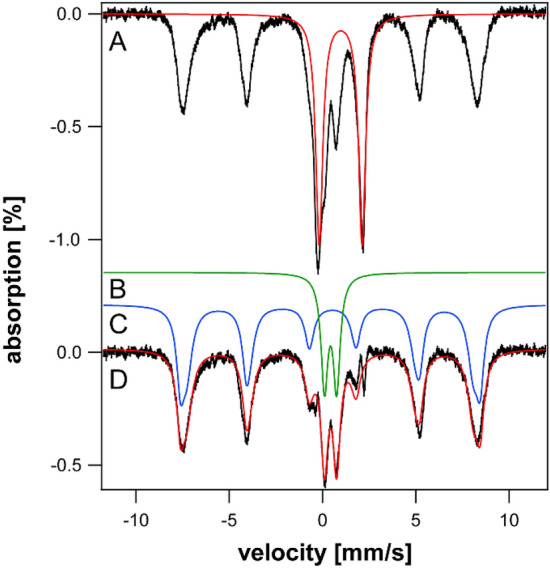
Mössbauer spectrum of spleen from 52 wk WT mice before (**A**) and after (D) removing blood contribution. The red line is a simulation of the standard blood quadrupole doublet, **B** green line is a simulation of the CD, and **C** blue line is a simulation of ferritin. Spectra were collected at 5–6 K and with 0.05 T field applied parallel to the gamma radiation unless mentioned otherwise

Our objective was to better define the iron content of iron-overloaded and diseased organs using MB spectroscopy, and to evaluate the age-dependence of overloading. The raw spectrum of every sample collected exhibited a quadrupole doublet arising from deoxy Fe^II^ hemoglobin. Table S1 includes parameters used for all simulations. The blood doublet was simulated as the red line in Fig. 1A. Blood contributed to all spectra despite perfusing animals extensively with buffer prior to dissection and rinsing the dissected organs with buffer prior to loading them into MB cups. A second blood doublet was evident exclusively in spectra of spleens from young mice. It had the same isomer shift but slightly smaller ΔE_Q_ (2.22 mm/s) than the primary blood doublet (2.32 mm/s). We suspect that it arose from fetal hemoglobin [49].

The percentage of raw spectral intensity due to the blood doublet varied from 5 to 90%. The absolute concentration of iron due to the blood was less than this range implies; an invariant concentration of blood iron affords a smaller percent of iron in spectra of overloaded organs relative to non-overloaded ones.

All other spectra presented below have had their hemoglobin contributions removed, and indicated percentages refer to hemoglobin-free difference spectra. With blood contributions removed, the dominant spectral feature for each organ was typically a magnetic sextet (Fig. 1C, blue line) arising either exclusively from ferritin or from ferritin and hemosiderin combined (see below). Also evident in most spectra was a central quadrupole doublet (called the *CD*) (Fig. 1B, green line) arising from a combination of [Fe_4_S_4_]^2+^ clusters and LS Fe^II^ hemes. Some samples exhibited a tiny-intensity quadrupole doublet due to non-heme-high-spin (NHHS) Fe^II^ species (not evident in Fig. 1).

### Spleen

The average mass of HFE spleens increased over the 52 wk duration of the study, from 55 → 180 mg (Table S2). The average mass of WT spleens at a similar age was slightly less. We removed the iron concentration due to blood from the overall HFE splenic iron concentrations, affording the values listed in Table S3. These concentrations were age-dependent, ranging from 850 µM in the spleen from a 6 wk mouse to 8300 µM in the spleen from a 52 wk mouse (*n* = 1 for each determination). These are likely to be the first absolute concentrations of splenic iron reported in which blood contributions were removed.

**Fig. 2:**
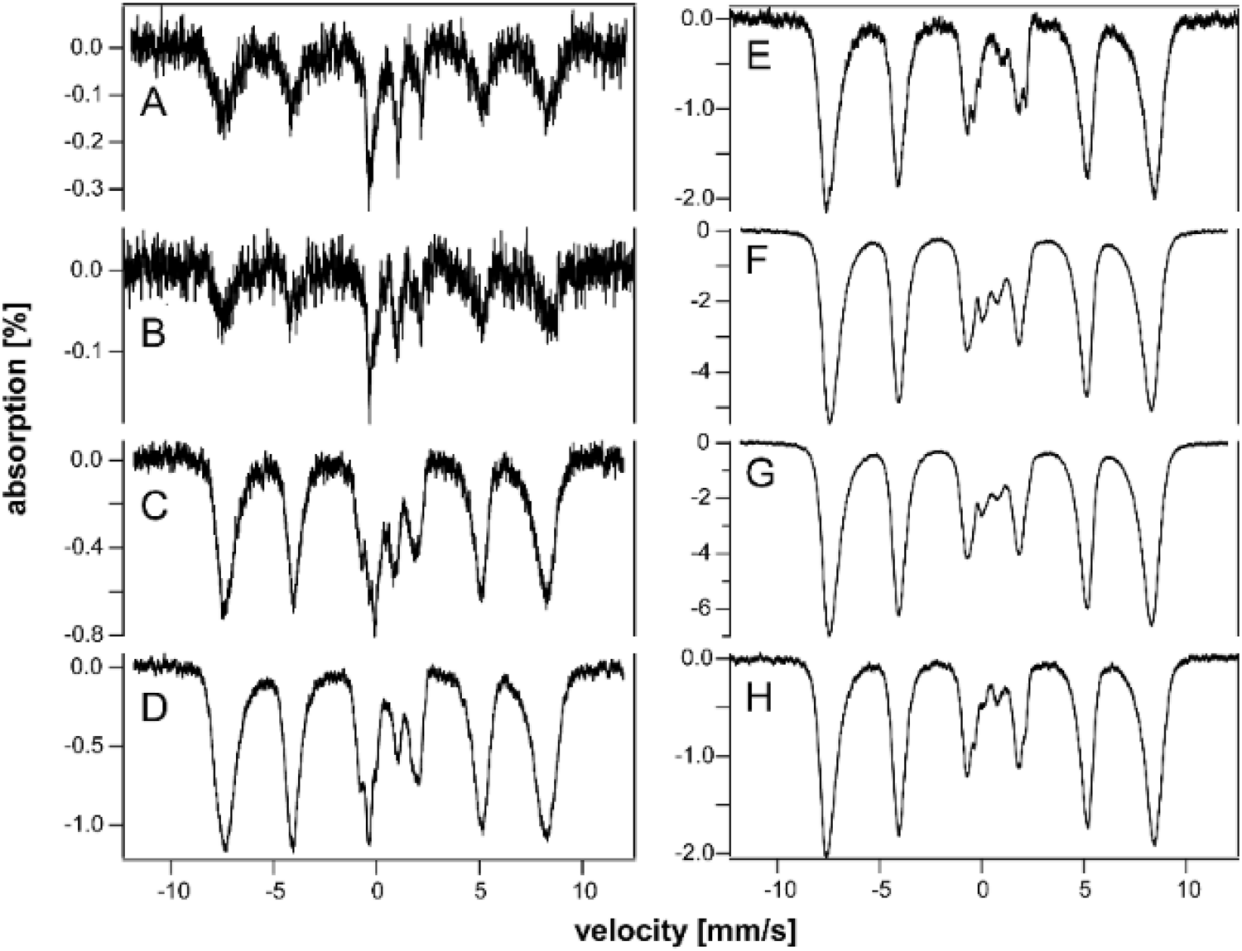
Mössbauer spectra of spleens isolated from HFE mice of different ages (in wk). **A** 3, **B** 4, **C** 10, **D** 14, **E** 18, **F** 20, **G** 32, and **H** 52

Most of the (non-hemoglobin) iron in spleens of young (3–4 wk) HFE mice was in the form of ferritin, with the CD representing just 15–30% of spectral intensity (Fig. 2, A and B). Starting at 4–10 wk of age, HFE spleens began accumulating even more ferritin (Fig. 2C–F), causing the CD to decline percentage-wise (all percentages are given in Table S4). By 32 and 52 wk, the concentration of iron in HFE spleens surpassed that in the liver while the CD declined to ~ 5% of spectral intensity (Fig. 2G and H). Whether this percentage-wise decline in the CD reflects an absolute decline in [Fe_4_S_4_]^2+^ clusters or LS Fe^II^ hemes is uncertain, but our limited data suggest that it does not.

**Fig. 3:**
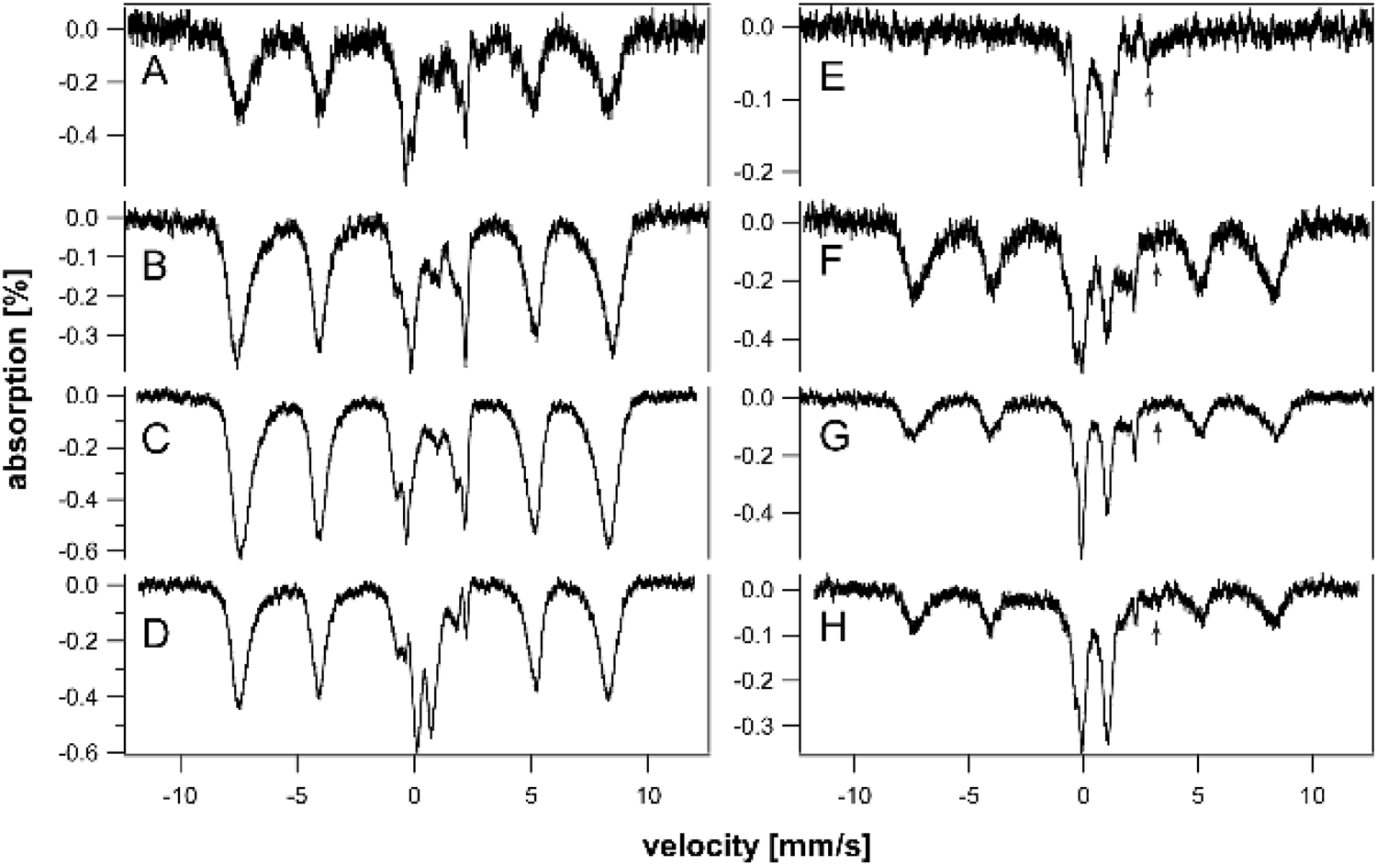
Mössbauer spectra of control spleens (**A**–**D**) and kidneys (**E**–**H**) isolated from WT control mice of different approximate ages. **A**, **B**, and **C**, “middle-aged”, **D** “old”. **E** “pups”, **F** and **G** “middle-aged”, **H** “old”. Arrows in **E**, **F**, and **G** may indicate a trace of NHHS Fe^II^

We identified four ^57^Fe-enriched spleens from WT mice in an earlier study conducted a decade ago. Spleens and kidneys were not the focus of those studies, but some specimens had been preserved in liquid N_2_. Spectra of spleens from WT mice labeled “middle-aged” and “old” (Fig. 3A–D) were also dominated by ferritin. These labels approximately translate to 6–35 and 35–96 wk, respectively; see [35–37]. In general, less iron accumulated in the spleens of control mice vs HFE spleens of approximately the same age. The “old” control spleen contained an unusual quadrupole doublet in the center of the spectrum that was absent in spectra of younger WT spleens. The parameters associated with it (δ = 0.38 mm/s and ΔE_Q_ = 0.9 – 1.0 mm/s) differed from those of the standard CD, and we did not assign it.

**Fig. 4:**
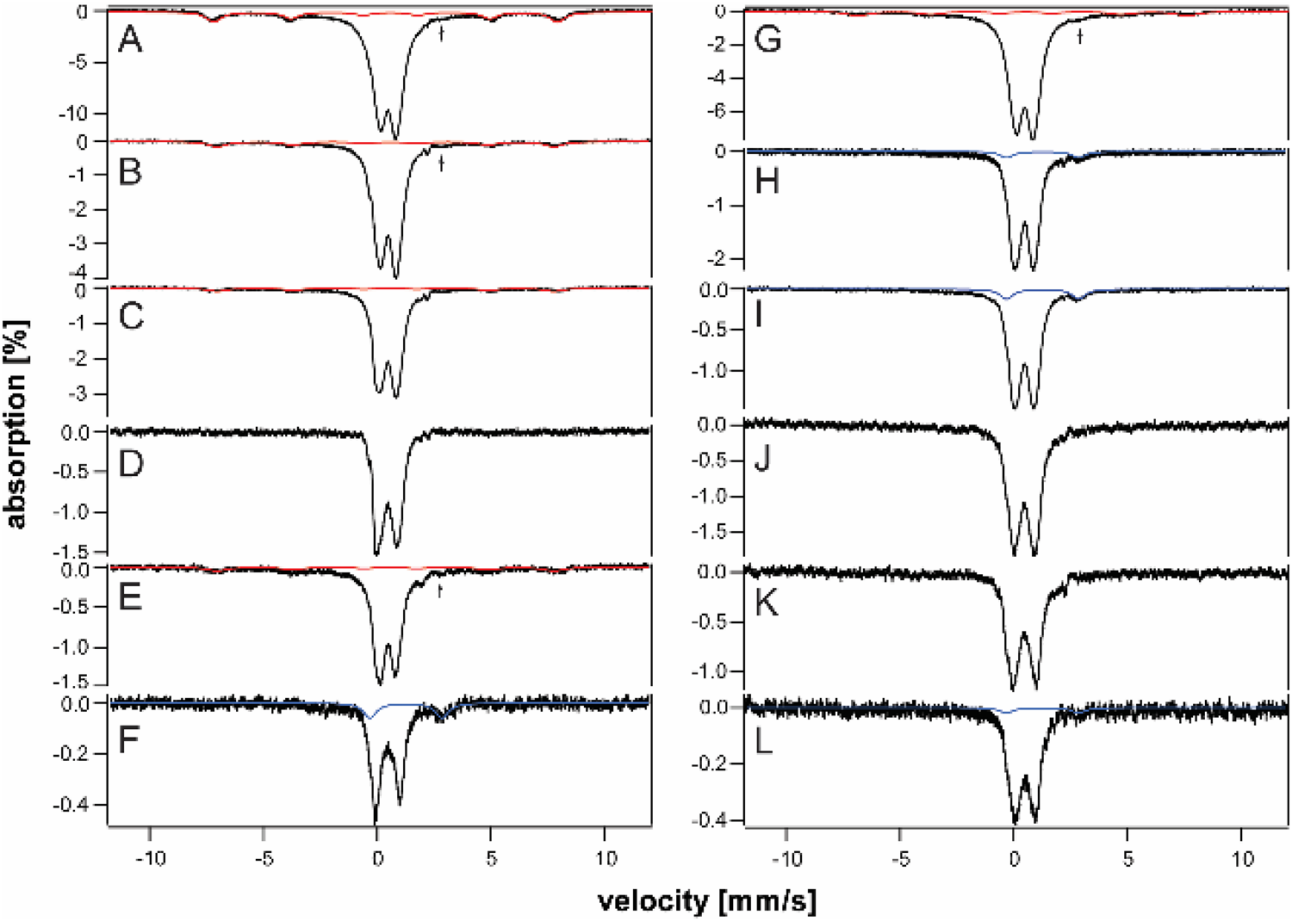
60 K Mössbauer spectra of organs. **A** 52 wk HFE spleen, **B** 20 wk HFE spleen, **C** 10 wk HFE spleen, **D** 6 wk HFE spleen, **E** “old” WT spleen, **F** “old” WT liver, **G** 52 wk HFE liver, **H** 32 wk HFE liver, **I** 20 wk HFE liver, **J** 52 wk HFE kidney, **K** 52 wk HFE heart, and **L** 52 wk HFE brain. Red lines are simulations of hemosiderin and blue lines are simulations of NHHS Fe^II^, both assuming parameters in Table S1. Arrows in A, B, E, and G indicate trace intensity due to NHHS FeII

To investigate the presence of hemosiderin, we collected 60 K spectra of HFE and control spleens of different ages (Fig. 4). Approximately 15- 20% of the iron in 10, 20, and 52 wk HFE mouse spleens exhibited magnetic sextet features originating from hemosiderin (Fig. 4A–C). Hemosiderin was also detected in control spleens (Fig. 4E) but not in HFE spleens at 6 wk (Fig. 4D). Some spleen spectra exhibited tiny absorption consistent with an NHHS Fe^II^ doublet (Fig. 4A, B, and E), but they represented only 1%—3% of total spectral intensity. Charitou et al. [50] reported NHHS Fe^II^ in the spleens of thalassemic (th3/ +) mice but none in controls.

### Liver

**Fig. 5:**
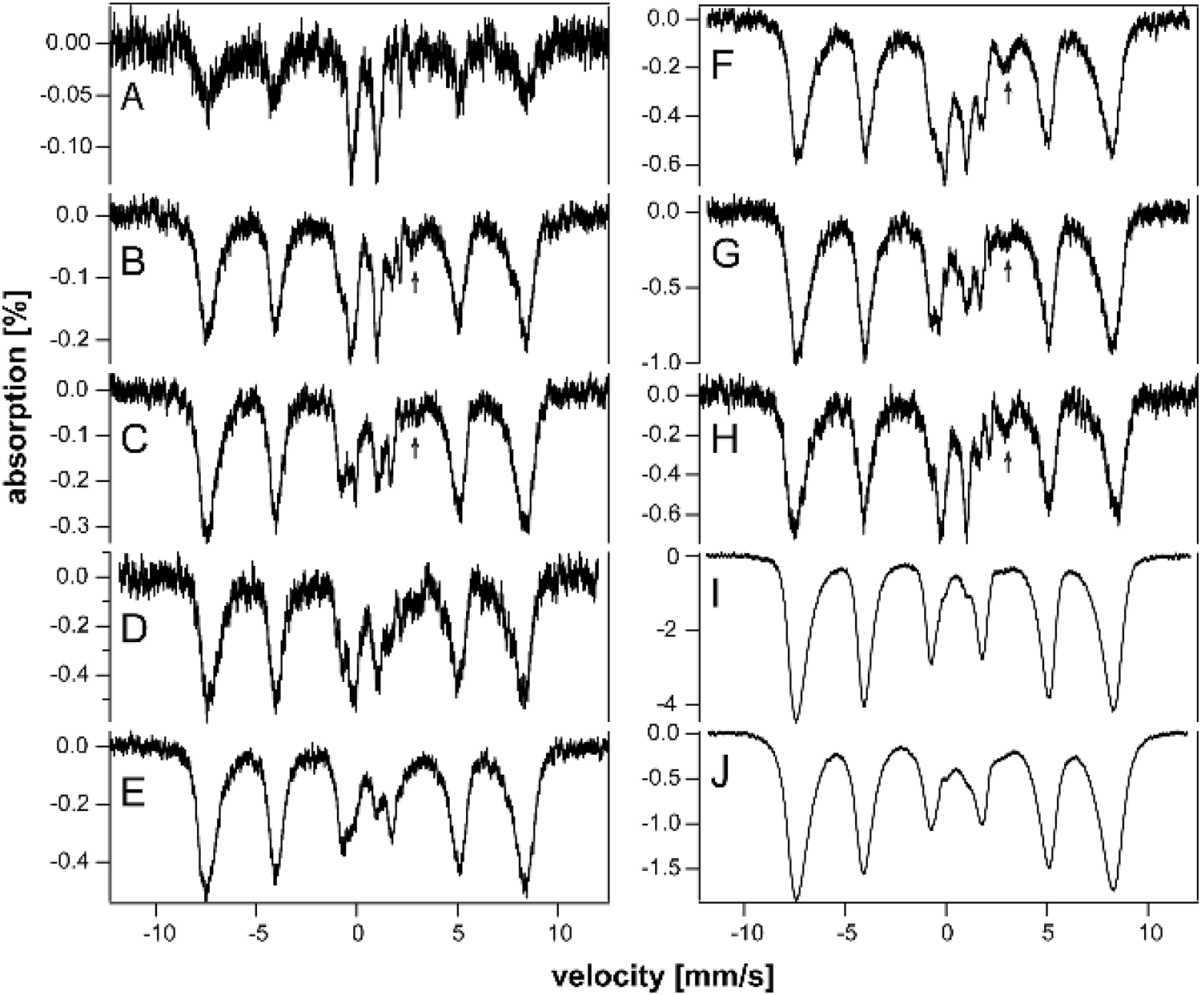
Mössbauer spectra of livers isolated from HFE mice of different ages (in wk). **A** 3, **B** 4, **C** 5, **D** 6, **E** 8, **F** 10, **G** 14, **H** 18, **I** 32, and **J** 52. Arrows in **B**, **C**, **F**, and **G** may be due to NHHS Fe^II^ doublets

Newborn livers from healthy control mice contain a high concentration of ferritin, but by 3 wk of age, most of the iron from ferritin exits the liver (Fig. 4C of [36]), leaving the CD dominating. At the same age, most iron in 3 wk HFE livers was ferritin, with ~ 40% CD (Fig. 5A). With each additional week of age, the spectral percentage due to ferritin increased, while that due to the CD declined (Table S4)—consistent with iron accumulating in HFE livers as ferritin. In terms of absolute concentration of iron, we estimated the concentration of CD iron in 20 wk HFE liver to be a few 100 µM, whereas that for ferritin was over 1500 µM. We previously estimated CD concentrations of ~ 180 µM in healthy livers (Table S2 of [47]) but only 30 µM in 12 wk HFE livers [37]. The discrepancy in CD concentrations may be due to uncertainties caused by spectral features of ferritin overlapping the CD.

The overall concentration of iron in HFE livers increased with age (Table S4). Based on spectral intensities, the loading of ferritin iron was roughly proportional to the animal’s age up until ~ 18 wk. Then, between 18 and 32 wk, there was a disproportionate increase in liver iron which was maintained to 52 wk, the end of the study. With age, the fraction of spectral intensity due to the CD declined, reaching only ~ 3% in the spectrum of the 52 wk old liver. The percent absorption is approximately proportional to the concentration of iron in the samples (since all MB cups were filled with liver tissue). Consistent with this, older HFE livers exhibited bronzing, whereas control livers did not. The proportion of liver iron due to ferritin also increased in control livers, but at a slower rate. Impressively, the spectrum of the HFE liver at 3 wk was most like that of a 96 wk control liver; see Fig. 1C of [36]. In summary, the iron contents of HFE livers at any age were qualitatively like those of healthy livers; both were dominated by ferritin followed by the CD. However, the percentages of ferritin vs CD in HFE livers were more characteristic of healthy livers at an older age.

Difference spectra exhibited minor errors due to subtracting the blood quadrupole doublet using simulation lines generated assuming a Voigt lineshape (which is approximately but not precisely correct). Subtraction errors became more noticeable as the percentage spectral intensity of the blood doublet increased. The subtraction error was most obvious near the high-energy line of a nonheme high-spin Fe^II^ doublet. This made it challenging to establish the presence of such a species, especially for the spectra of Fig. 5A–E. A different challenge arose when minor spectral features became overwhelmed by the majority species; e.g., attempting to identify NHHS Fe^II^ features in the presence of intense ferritin features, as in Fig. 5I–J. Fortunately, there was a “window” associated with the spectra of Fig. 5F–H. Those spectra exhibited a high-energy line of a NHHS Fe^II^ doublet that was largely free from subtraction error and not overwhelmed by ferritin spectral features (Fig. 5, arrows).

We previously reported the absence of hemosiderin in a 12 wk liver from an HFE mouse, as judged from the absence of a sextet feature in the MB spectra at 70 K [37]. In the current study, we collected 60 K MB spectra of HFE livers of three different ages. About 20% of the spectral intensity from of a 52 wk old HFE liver was due to hemosiderin (Fig. 4G), whereas spectra of a 32 wk liver and 20 wk HFE liver were devoid of hemosiderin (Fig. 4H and I), as was the liver from a 96 wk control mouse [36]. The high-energy line of the NHHS Fe^II^ doublet was clearly present in the 32 and 20 wk spectra. A similar feature was present in the spectrum of the 52 wk liver (Fig. 4G) but is less evident, because the collapsed ferritin features dominated. Livers examined from healthy control mice were devoid of hemosiderin [37]. Charitou et al. [50] previously reported the absence of hemosiderin in livers from th3/ + mice.

### Kidneys

**Fig. 6:**
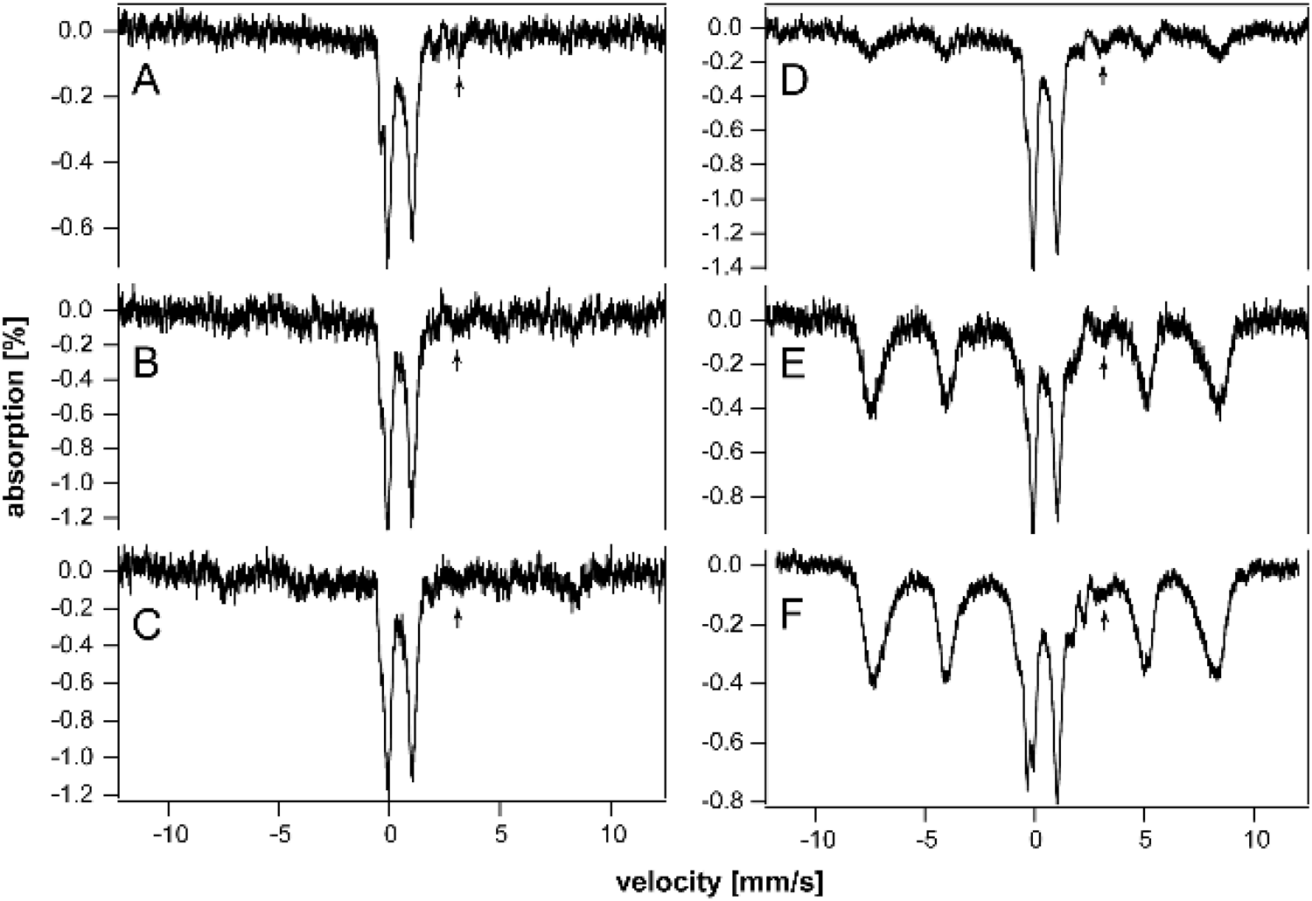
Mössbauer spectra of kidneys isolated from HFE mice of different ages (in wk). **A** 3, **B** 10, **C** 14, **D** 18, **E** 32, and **F** 52 wk. Arrows indicate NHHS Fe^II^ doublet

Mössbauer spectra of young and middle-aged HFE kidneys (3–14 wk) were dominated by the CD and were virtually devoid of ferritin iron (Fig. 6A–C). At 18 wk (Fig. 6D), ferritin iron began developing intensity, and by 32 and 52 wk (Fig. 6E and F), it dominated the spectra. Unlike spectra of other HFE organs, the CD intensity remained intense even for older animals. The spectrum of control kidneys labeled “pups” was also dominated by the CD and was devoid of ferritin (Fig. 3E). The spectra of “middle aged” control kidneys (Fig. 3F and G) were indistinguishable from their HFE counterparts; all contained significant spectral percentages of both CD and ferritin. The spectrum of “old” WT control kidney (Fig. 3H) was similar to HFE kidneys from 32 wk mice, whereas kidneys from 52 wk HFE mice contained a higher percentage of ferritin. Unlike other organs, the iron contents of kidneys from HFE mice at all ages were indistinguishable (at this level of analysis and between 3 to 52 wk of age) from WT controls. This suggests that the ability to store iron as ferritin is more limited in kidneys than in livers or spleens. The non-hemoglobin iron concentrations of HFE kidneys increased slightly with age, from 160 µM (for a 4 wk sample) to 560 µM (at 32 and 52 wk) (Table S4). A spectrum of the 52 wk HFE kidney collected at 60 K (Fig. 4J) was devoid of a sextet that would have indicated hemosiderin; nor did it exhibit an NHHS Fe^II^ doublet. In contrast, Charitou et al. [50] reported 500% more NHHS Fe^II^ in the kidneys of thalassemic (th3/ +) mice compared to controls.

### Heart and brain

**Fig. 7:**
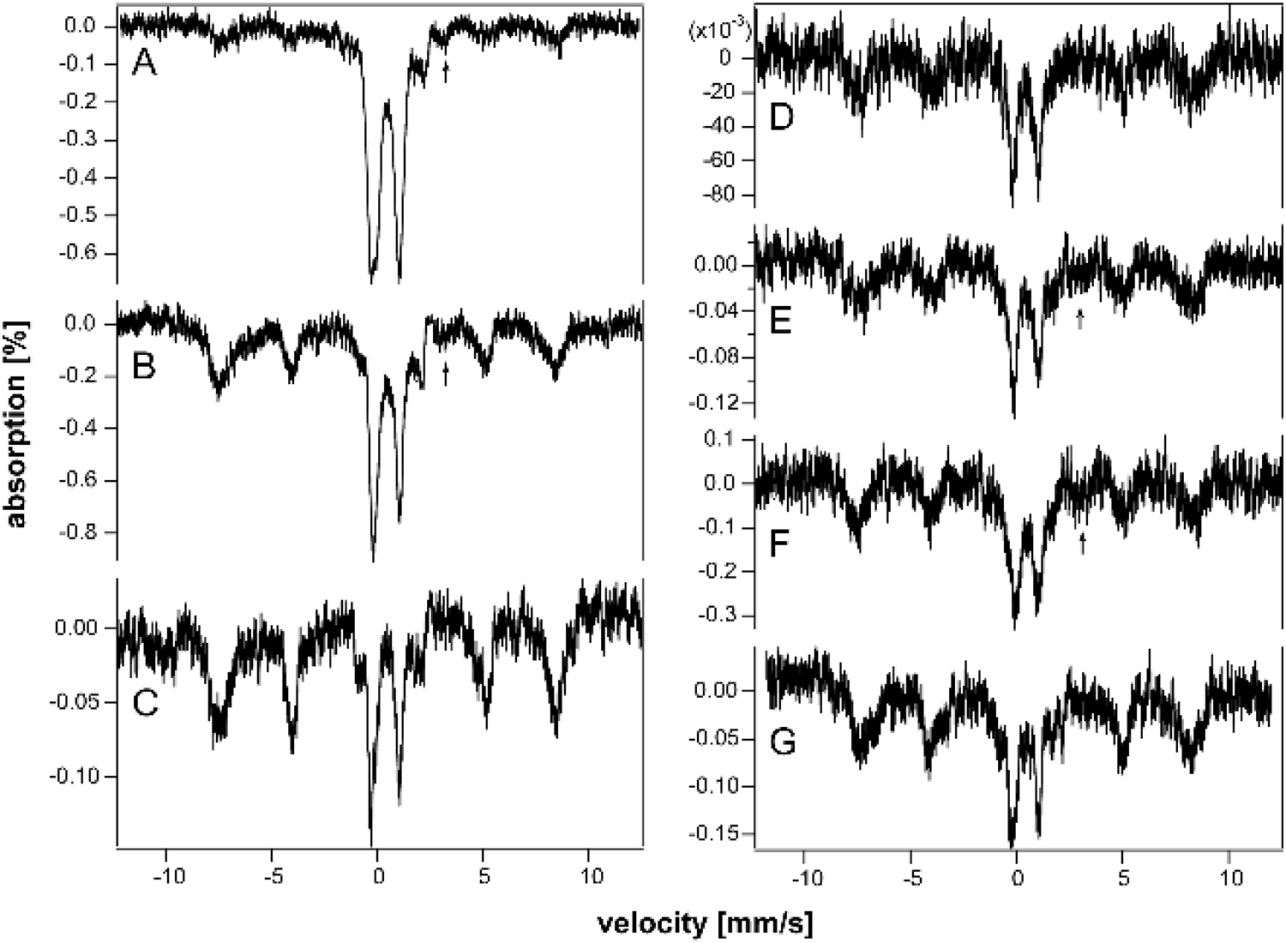
Mössbauer spectra of hearts (**A**–**C**) and brains (**D**–**G**) isolated from HFE mice of different ages (in wk). **A** 3, **B** 10, **C** 52, **D** 3, **E** 4, **F** 10, and **G** 52. Arrows may indicate NHHS Fe^II^ doublets

HFE hearts did not accumulate nearly as much ferritin as did livers and spleens (Fig. 7A–C). On the other hand, they accumulated more iron than WT control hearts in the same age group. The spectrum of a 3 wk HFE heart (Fig. 7A) exhibited more ferritin-based features than those of control WT hearts between the age of newborn and 4 wk (See Fig. 2 of [37]). That of an HFE heart at 10 wk (Fig. 7B) also exhibited a greater percentage of ferritin than adult controls (see Fig. 3 of [37]). The spectrum of a 52 wk HFE heart (Fig. 7C) was similar to that of a 60 wk control heart (Fig. 1D of [37]). MB spectra of hearts from young healthy controls (up to 4 wk of age) were dominated by the CD and contained only ~ 18% ferritin on average (Fig. 2 of [37]). Corresponding spectra from adult control mice (6–28 wk) were also dominated by the CD but contained ~ 46% ferritin. Older control mice contained ~ 70% ferritin with the remainder in the form of the CD. In contrast, hearts from 3, 18, and 52 wk old HFE mice contained 20, 65, and 80% ferritin, respectively (Table S4). Thus, using the fraction of ferritin loading in the heart, hearts of HFE mice appeared “older” than their chronological age relative to controls. The 60 K MB spectrum of 52 wk hearts (Fig. 4K) was devoid of any magnetic feature, indicating the absence of hemosiderin.

The brain contains little iron, and so, MB spectral intensities were weak (Fig. 7D–G). Within that constraint, there were no major differences evident between the HFE and control brains in similarly aged mice (Fig. 1 of [35]). There was slightly more ferritin in HFE brains at the same age. For control brains, there was no trend from 3, 4, 24, and 58 wk, with an average of 53% ferritin and 34% CD [35]. The 60 K MB spectrum of 52 wk HFE brains (Fig. 4L) was devoid of any magnetic feature, indicating the absence of hemosiderin. There was perhaps a hint of NHHS Fe^II^. Charitou et al. [50] reported that brains from 9 month thalassemic mice contained around 10% more ferritin than WT mice, comparable to what we observed in HFE brains.

## Discussion

### Age-dependence of iron-overload in HFE organs

The iron content of all investigated HFE organs, as determined by low-temperature MB spectroscopy, was similar to the same healthy organ but from an older mouse. HFE organs accumulated more ferritin than controls, and they did so starting at an earlier age. The liver and spleen were the first to be overloaded, followed by the kidney, and then, to a lesser extent, heart and brain. Although these latter two organs were less overloaded, even minor overloading might still affect their physiological function.

Previous studies have not observed iron-overloaded spleens in HFE mice. Zhou et al. [51] reported that spleens of 10 wk HFE mice were not iron-overloaded. Albalat et al. [52] reported a similar result with 12 month old HFE mice. Cavey et al. [53] saw splenic iron-overload but only in WT mice in which iron-overload was induced dietarily; no overload was observed in HFE mice. We cannot explain this apparent discrepancy with previous studies. However, there is no doubt that we have observed iron-overloaded spleens. Although we only have one Mössbauer spectrum for each age reported in Fig. 2, each sample contained 2–3 spleens, and so, the spectra represent an average.

**Fig. 8:**
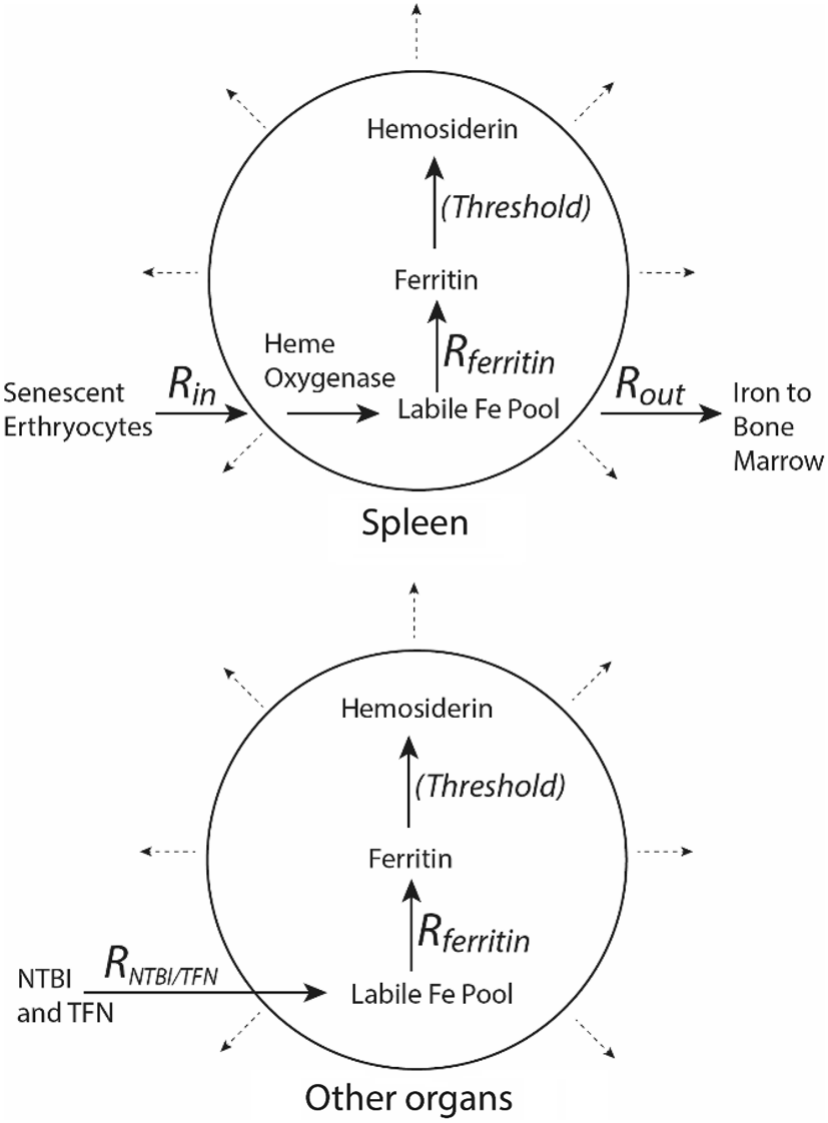
Model of iron-overloading in the spleen (top) and other organs (bottom). Cell growth is indicated by the dashed arrows

Our general understanding of iron-overload in spleen and other organs is illustrated in Fig. 8. The rate of iron import (*R*_*in*_), which is mainly associated with red-cell recycling in spleen, is counterbalanced by the rates of iron export *R*_*out*_ (to bone marrow for erythropoiesis) and dilution due to cell/organ growth (*R*_*dil*_). For iron to accumulate in the spleen, *R*_*in*_ might increase relative to its rate under WT conditions, *R*_*out*_ might decrease, or *R*_*dil*_ might decrease (or some combination of these). Cavey et al. [54] found that the concentration of erythrocytes in HFE mice is ~ 6% higher than in controls, suggesting an increased *R*_*in*_. Organ growth declines as animals’ age, and so, *R*_*dil*_ might have also decreased with age. In principle, *R*_*out*_ should *increase* in HFE mice, relative to the WT state (due to less hepcidin and more FPN), causing iron depletion. Since iron-overload was observed, this effect may not dominate under the conditions examined. The excess iron in the spleen first accumulates as ferritin, indicating an increased rate of ferritin metallation (*R*_*ferritin*_). The iron used for this process is likely obtained from the degradation of heme as catalyzed by heme oxygenase [55]. The mechanism explaining the accumulation of ferritin iron in other organs (Fig. 8, bottom) is assumed to be similar except that these organs import either transferrin or NTBI rather than senescent erythrocytes, and they may not import or export iron as quickly as in the spleen.

### Iron-dependent threshold activation of ferritin degradation

This was our first study in which we detected hemosiderin in any sample; here, we detected it in spleens and livers of older HFE mice, as well as in the spleen of an older control mouse. In all cases, the concentration of ferritin in the organ was high (2–3 mM Fe or higher). The percent of iron in the form of hemosiderin never exceeded ~ 20%—i.e., ferritin still dominated. Consistent with this, Charitou et al. [50] also observed hemosiderin in iron-overloaded spleens, in their case from th3/ + (heterozygous β-thalessemia) mice. They also reported that ferritin rather than hemosiderin dominated the iron content of iron-overloaded spleens; hemosiderin represented ~ 19 and 23% of total splenic iron at 6 and 9 months of age, respectively (our estimates), similar to our observations.

Earlier studies have reported hemosiderin in diseased iron-overloaded organs—but they also reported that it exceeded ferritin (see Introduction). One possible explanation for this discrepancy is that earlier samples might have been more overloaded and thus had a higher Fe concentration. Such samples were routinely *not* enriched in ^57^Fe, so perhaps samples with exceedingly high iron concentrations were selected to achieve the highest possible signal/noise ratios in Mössbauer spectra. Also, the mice we used (and those used by Charitou et al. [50]) were fed only 50 mg ^57^Fe per kg chow—which is ca. 1/5th of the iron in normal chow. This could have moderated the extent of hemosiderin generation. Freeze-thawing had no effect on the ferritin/hemosiderin ratio [56]. Another concern with those earlier studies is that typically only high-temperature spectra were collected which complicates interpretations.

We propose that the degradation of ferritin → hemosiderin becomes activated only after a threshold concentration of iron (or perhaps only of ferritin) has been is exceeded. We estimate the threshold to be ca. 2–3 mM total (non-hemoglobin) iron. In our samples, that threshold was exceeded in both spleen and liver, and especially (but not exclusively) in older HFE samples. The concentration of ferritin in spleen was actually greater than in liver (Table S3). This difference is even more extreme than reflected in % spectral absorption, because the liver samples completely filled the MB cup, whereas spleen samples occupied only ~ half of the cup volume.

### “Surface-bound ferritin” spectral feature originates from [Fe_4_S_4_]^2+^ clusters and LS Fe^II^ hemes

Charitou et al. [50] enriched th3/ + and WT mice with ^57^Fe, and collected hearts, livers, kidneys, brains, and spleens at 1, 3, 6, and 9 months (with 1–2 mice of each strain per time-point). MB spectra at 80 K exhibited a blood doublet, a nonheme high-spin Fe^II^ doublet, and two additional doublets. Following Bou-Abdalla et al. [57], one of the additional doublets (*δ* = 0.46 mm/s and ΔEQ = 0.59 mm/s) was assigned to the inner core of ferritin, and the other (*δ* = 0.46 mm/s and ΔE_Q_ = 1.05 mm/s) was assigned to the surface irons of the ferritin core. The parameters of the surface-ferritin doublet are remarkably similar to those of the CD in our spectra. We hypothesize that the two doublets arise from the same species, namely diamagnetic [Fe_4_S_4_]^2+^ clusters and low-spin ferrous heme centers. At 80 K, MB spectra cannot distinguish superparamagnetic ferritin from these diamagnetic centers as both would yield quadrupole doublets. However, the distinction is clear in our spectra collected at ~ 5 K, because ferritin and hemosiderin exhibit magnetic sextets at that temperature, whereas diamagnetic *S* = 0 [Fe_4_S_4_]^2+^ clusters, [Fe_2_S_2_]^2+^ clusters, and *S* = 0 Fe^II^ hemes exhibit quadrupole doublets devoid of magnetic hyperfine interactions.

This observed behavior unambiguously supports our assignment of the disputed doublet. Reinforcing our assignment, many other results of Charitou et al. would agree fully with ours if the “surface-ferritin” doublet were reassigned to the CD and “inner-ferritin” were assigned to both surface and core ferritin iron combined. For example, Charitou et al. [50] found that the ratio of the inner/surface-ferritin doublets increased with age and differed between organs, in agreement with our observations. In WT hearts, kidneys, and brain, “surface ferritin” (i.e., the CD) dominated, whereas in the spleen and liver, “inner ferritin” (i.e., ferritin) dominated, as we observed. With age, the proportion of iron due to ferritin (or inner ferritin) increased, also as we observed. We both observed modest spectral differences between Th3/ + and WT hearts. We both observed major iron accumulation in the liver (4–9.5 × more ferritin in Th3/ + adults than in controls). For kidneys, Charitou et al. observed an increase in ferritin at 12 wk, similar to the increase we observed at 15 wk. We both observed slight ferritin accumulation in the brain.

### MB spectroscopy is complementary to NMR

NMR has the huge advantage of being non-invasive and performable at room-temperature and on live patients [58]. The major advantage of MB spectroscopy is that it detects all forms of iron, including diamagnetic iron. Moreover, relative MB spectral intensities are approximately proportional to the concentrations of each species in the sample. NMR primarily detects ferritin and cannot detect diamagnetic iron centers, including oxidized ISCs and low-spin Fe^II^ hemes; it is sensitive only to the overall effect of iron-associated paramagnetism on proton relaxation rates. Thus, NMR seems unable to easily distinguish ferritin from hemosiderin. Magnetic susceptibility suffers from similar problems.

All problems considered, low-temperature MB studies of iron-overloaded ^57^Fe-enriched mouse organs provide the most rigorous description for decomposing iron (overloaded or not) in mammals. Such studies could be performed on any genetic strain of mice, and in an age-dependent manner, to evaluate how iron contents are changing. Much remains to be learned as to the mechanism of hemosiderin formation. Performing NMR on matched ^57^Fe-enriched mouse organs could allow MB to calibrate and interpret the iron content of NMR images of comparable organs from human patients. This combination of spectroscopic methods might offer a distinct advantage in understanding the process of iron-overloading and in treating iron-overload diseases.

## Supplementary Information

Below is the link to the electronic supplementary material.

Supplementary file 1 (DOCX 50 KB)

## Data Availability

Spectra available upon request.

## Abbreviations

CD: Central doublet
FRN: Ferritin
HFE: Homeostatic Fe regulator gene
ISC: Iron–sulfur cluster
MB: Mössbauer
NHHS: Non-heme high-spin
NTBI: Non-transferrin-bound iron
TAMU: Texas A&M University
th3/ +: Thalassemic mice
TFN: Transferrin
WT: Wild type
