# Supplementary material for: Low-temperature Mössbauer spectroscopy of organs from 57Fe-enriched HFE(−/−) hemochromatosis mice: an iron-dependent threshold for generating hemosiderin
Source: J Biol Inorg Chem. 2022 Dec 13;28(2):173–85. doi: 10.1007/s00775-022-01975-y (PMC9981716; doi:10.1007/s00775-022-01975-y)
Supplement: Supplementary file 1 — (DOCX 50 KB) [file 775_2022_1975_MOESM1_ESM.docx]

**Supplemental Information**

Title: Low-temperature Mössbauer spectroscopy of organs from ^57^Fe-enriched HFE^(-/-)^ hemochromatosis mice: an iron-dependent threshold for hemosiderin generation

Journal: Journal of Biological Inorganic Chemistry

Authors: Shaik Waseem Vali and Paul A. Lindahl

Affiliation: Departments of Biochemistry and Biophysics, and of Chemistry, Texas A&M University, College Station, TX 77843-3255 USA.

Email address of the corresponding author: [Lindahl@chem.tamu.edu](mailto:Lindahl@chem.tamu.edu)

**Table of Contents:**

Table S1: Mössbauer parameters used in fitting.

Table S2: Average mass of spleens in HFE and WT mice.

Table S3: Iron concentrations in ^57^Fe-enriched HFE organs after removing blood contributions.

Table S4: Spectral Percentages of ferritin (FTN) and the central doublet (CD) used in simulations.

**Table S1: Mössbauer parameters used in fitting.**

|  | Central Doublet | Ferritin | Blood |
| --- | --- | --- | --- |
| Isomer Shift (δ) (mm/s) | 0.42±0.02  0.37±0.02 (Fig 3 D) | 0.46±0.06 | 0.93±0.02  0.92±0.02 (Fig 2 A-D) |
| Quadrupole Splitting (ΔE_Q_) (mm/s) | 1.1±0.05  0.85±0.05 (Fig 3 D) | -0.2±0.08 | 2.32±0.03  2.20±0.02 (Fig 2 A-D) |
| Linewidth (Γ) (mm/s) | 0.45±0.04 | 0.6±0.15 | 0.38±0.02 |
| H_eff_ (kG) |  | 480±10 |  |

**Table S2: Average mass of spleens in HFE and WT mice.**

| Strain | Age (wk) | Replicates (n) | Average spleen mass (g) |
| --- | --- | --- | --- |
| HFE (this study) | 4 | 6 | 0.055 ± 0.018 |
| HFE | 6 | 8 | 0.071 ± 0.019 |
| HFE | 12 | 8 | 0.086 ± 0.037 |
| HFE | 20 | 8 | 0.109 ± 0.031 |
| HFE | 24 | 8 | 0.115 ± 0.034 |
| HFE | 32 | 6 | 0.158 ± 0.033 |
| HFE | 56 | 4 | 0.181 ± 0.053 |
|  |  |  |  |
| WT (this study) | 12 | 10 | 0.103 ± 0.036 |
| WT | 16 | 7 | 0.133 ± 0.056 |
| WT | 24 | 9 | 0.120 ± 0.061 |
| WT | 32 | 8 | 0.133 ± 0.073 |

**Table S3: Iron concentrations in ^57^Fe-enriched HFE organs after removing blood contributions.** Blood contributions were determined from Mössbauer spectral intensities. Entries are singletons, with uncertainties arising from 3 technical replicates.

| Organ | Age (wk) | Blood iron (%) | [Fe] (µM) |
| --- | --- | --- | --- |
| Liver | 20 | 23 | 1800 ± 30 |
| Liver | 32 | 25 | 2400 ± 40 |
| Liver | 52 | 5 | 3200 ± 30 |
| Spleen | 6 | 55 | 850 ± 40 |
| Spleen | 10 | 30 | 2300 ± 40 |
| Spleen | 20 | 25 | 6600 ± 90 |
| Spleen | 52 | 10 | 8300 ± 80 |
| Kidney | 4 | 65 | 160 ± 20 |
| Kidney | 14 | 55 | 280 ± 30 |
| Kidney | 20 | 55 | 380 ± 30 |
| Kidney | 32 | 40 | 550 ± 20 |
| Kidney | 52 | 28 | 560 ± 30 |

**Table S4: Spectral Percentages of ferritin (FTN) and the central doublet (CD) used in simulations.** In some spectra, a minor contribution of the magnetic sextet assigned to ferritin was due to hemosiderin (see below). NHHS Fe^II^ doublets typically accounted for 0-2% and were not routinely included in simulations. Uncertainties are estimated at ± 5%.

| Strain | Age (wk) | Liver (Fig 5) | | Spleen (Fig. 2) | | Kidney (Fig. 6) | | Heart (Fig. 7) | | Brain (Fig. 7) | |
| --- | --- | --- | --- | --- | --- | --- | --- | --- | --- | --- | --- |
|  |  | CD | FTN | CD | FTN | CD | FTN | CD | FTN | CD | FTN |
| HFE | 3 | 25 | 75 | 20 | 80 | 100 | - | 80 | 20 | 25 | 75 |
| HFE | 4 | 20 | 80 | 20 | 80 |  |  |  |  | 25 | 75 |
| HFE | 5 | 10 | 90 |  |  |  |  |  |  |  |  |
| HFE | 6 | 15 | 85 |  |  |  |  |  |  |  |  |
| HFE | 10 | 10 | 90 | 15 | 85 | 95 | 5 |  |  |  |  |
| HFE | 14 | 15 | 85 | 10 | 90 | 90 | 10 |  |  |  |  |
| HFE | 18 | 10 | 90 | 5 | 95 | 75 | 25 | 35 | 65 | 25 | 75 |
| HFE | 20 | 15 | 85 | 5 | 95 |  |  |  |  |  |  |
| HFE | 32 | <5 | >95 | 5 | 95 | 25 | 75 |  |  |  |  |
| HFE | 52 | <5 | >95 | <5 | >95 | 20 | 80 | 20 | 80 | 20 | 80 |
| Control | Pups |  |  |  |  | >95 (Fig 3) | <5 (Fig 3) |  |  |  |  |
| Control | middle aged |  |  | 5 (Fig 3) | 95 (Fig 3) | 20-30 (Fig 3) | 75-85 (Fig 3) |  |  |  |  |
| Control | old |  |  | 15 (Fig 3) | 85 (Fig 3) | 35 (Fig 3) | 65 (Fig 3) |  |  |  |  |
